# Metagenomic Next-Generation Sequencing for Identification and Quantitation of Transplant-Related DNA Viruses

**DOI:** 10.1128/JCM.01113-19

**Published:** 2019-11-22

**Authors:** Meredith L. Carpenter, Susanna K. Tan, Thomas Watson, Rowena Bacher, Vaishnavi Nagesh, Alain Watts, Gordon Bentley, Jenna Weber, ChunHong Huang, Malaya K. Sahoo, Armin Hinterwirth, Thuy Doan, Theodore Carter, Queeny Dong, Stéphane Gourguechon, Eric Harness, Sean Kermes, Srihari Radhakrishnan, Gongbo Wang, Alejandro Quiroz-Zárate, Jesus Ching, Benjamin A. Pinsky

**Affiliations:** a Arc Bio, LLC, Scotts Valley, California, USA; b Arc Bio, LLC, Cambridge, Massachusetts, USA; c Department of Medicine, Division of Infectious Diseases and Geographic Medicine, Stanford University School of Medicine, Stanford, California, USA; d Department of Pathology, Stanford University School of Medicine, Stanford, California, USA; e Francis I Proctor Foundation, University of California San Francisco, San Francisco, California, USA; f Department of Ophthalmology, University of California San Francisco, San Francisco, California, USA; University Hospital Münster

**Keywords:** DNA sequencing, genomics, virology, transplant infectious diseases

## Abstract

Infections with DNA viruses are frequent causes of morbidity and mortality in transplant recipients. This study describes the analytical and clinical performance characteristics of the Arc Bio Galileo Pathogen Solution, an all-inclusive metagenomic next-generation sequencing (mNGS) reagent and bioinformatics pipeline that allows the simultaneous quantitation of 10 transplant-related double-stranded DNA (dsDNA) viruses (adenovirus [ADV], BK virus [BKV], cytomegalovirus [CMV], Epstein-Barr virus [EBV], human herpesvirus 6A [HHV-6A], HHV-6B, herpes simplex virus 1 [HSV-1], HSV-2, JC virus [JCV], and varicella-zoster virus [VZV]). The mNGS 95% limit of detection ranged from 14 copies/ml (HHV-6) to 191 copies/ml (BKV), and the lower limit of quantitation ranged from 442 international units (IU)/ml (EBV) to 661 copies/ml (VZV). An evaluation of 50 residual plasma samples with at least one DNA virus detected in prior clinical testing showed a total percent agreement of mNGS and quantitative PCR (qPCR) of 89.2% (306/343), with a κ statistic of 0.725. The positive percent agreement was 84.9% (73/86), and the negative percent agreement was 90.7% (233/257). Furthermore, mNGS detected seven subsequently confirmed coinfections that were not initially requested by qPCR. Passing-Bablok regression revealed a regression line of *y* = 0.953*x* + 0.075 (95% confidence interval [CI] of the slope, 0.883 to 1.011; intercept, −0.100 to 0.299), and Bland-Altman analysis (mNGS − qPCR) showed a slight positive bias (0.28 log_10_ concentration; 95% limits of agreement, −0.62 to 1.18). In conclusion, the mNGS-based Galileo pipeline demonstrates analytical and clinical performance comparable to that of qPCR for transplant-related DNA viruses.

## Abstract (precis)

Infections with DNA viruses are frequent causes of morbidity and mortality in transplant recipients.

## INTRODUCTION

Solid organ transplant (SOT) and hematopoietic cell transplant (HCT) recipients are uniquely susceptible to infection, often with increased severity due to a number of common and opportunistic viruses. Specifically, viral infections with human adenovirus (ADV), cytomegalovirus (CMV), Epstein-Barr virus (EBV), BK virus (BKV), human herpesvirus 6A and -B (HHV-6A and HHV-6B, respectively), JC virus (JCV), varicella-zoster virus (VZV), and herpes simplex virus 1 and -2 (HSV-1 and -2, respectively) can result in graft failure and even death (1–4). These infections can be derived from reactivation of latent virus, transmission of the virus from the transplant, or primary infection (1). For example, CMV is an important cause of posttransplant tissue-invasive disease, particularly of the gastrointestinal and respiratory tracts (1, 5–8), EBV drives the development of posttransplant lymphoproliferative disorders (9, 10), and BKV causes nephropathy, a serious complication following renal transplantation (11).

These viruses are regularly diagnosed and monitored in transplant recipients in order to assess for the risk or progression of disease, initiate preemptive or symptomatic therapy, and determine the efficacy of direct antiviral agents and/or the reduction of immunosuppression (12). The majority of the transplant viral load testing in clinical laboratories utilizes real-time, quantitative PCR (qPCR) assays targeting the virus of interest calibrated to copies or international units (IU)/ml plasma, depending on the virus (13–20). Though coinfections are common in transplant recipients (21–23), the potential for virus at high levels to outcompete virus at lower, but still clinically significant, levels in single-tube, multiplex PCRs results in transplant viral load monitoring being performed one virus at a time.

Metagenomics analysis using next-generation sequencing (mNGS) is a promising approach to determine the presence and abundance of transplant-related viral infections, as well as identify coinfections in an unbiased manner (24). However, while clinical microbiology and virology laboratories have widely embraced quantitative molecular methods, NGS has not yet been broadly adopted due to its high cost per sample, long turnaround time, and the lack of technical and computational expertise required to produce and analyze the data.

**FIG 1:**
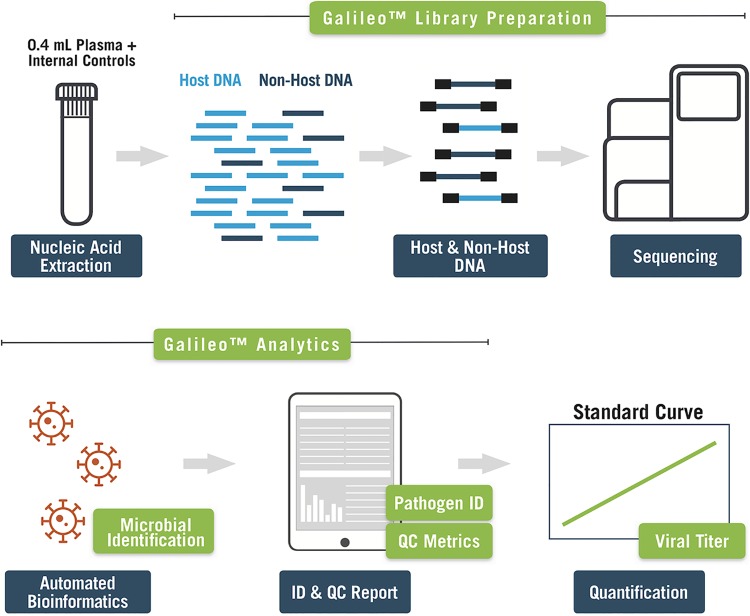
Overview of the Galileo Pathogen Solution pipeline. Plasma is extracted and converted into a next-generation sequencing library using the Galileo library preparation kit, which includes external and internal full-process controls, library preparation reagents, and dual-indexed adapters. Following sequencing, the Galileo Analytics automated informatics pipeline produces quality control and pathogen identification reports, and a standard curve is used to determine viral load values.

A recently developed mNGS approach for the quantitation of transplant-related DNA viruses is the Galileo Pathogen Solution, a product commercialized by Arc Bio, LLC, in 2019. Galileo comprises a suite of reagents and software that can be used to sequence pathogen nucleic acids from plasma, including internal full-process controls, external run controls, and a cloud-based Web application that enables virus identification and quantitation (Fig. 1). In this study, the analytical characteristics of the Galileo pipeline were investigated using an initial set of 10 transplant-associated DNA viruses, and its clinical performance was compared to that of qPCR on clinical samples from immunocompromised patients with known viremia.

## MATERIALS AND METHODS

### Ethics statement.

This study was reviewed and approved by the institutional review board of Stanford University (protocol no. IRB-32934).

### Reference viruses.

The analytical experiments were performed by spiking a multianalyte mixture of whole-virus particles composed of 10 viruses (CMV strain AD169, EBV strain B95, ADV type 1, BKV subtype 1b-2, JCV type 1a, HHV-6A strain GS, HHV-6B strain Z-29, HSV-1 strain 95, HSV-2 strain 09, and VZV strain 9/84; Arc Bio, LLC) into negative human plasma screened for the target viruses by both Galileo (Arc Bio, LLC, and Stanford) and qPCR (Stanford). Viral loads are reported in standardized international units per milliliter where available (CMV, EBV, and BKV); otherwise, viral loads are reported in genome copies per milliliter.

### Clinical samples.

Inclusion criteria for the clinical plasma specimens were the presence of at least one transplant-related DNA virus (ADV, BKV, CMV, EBV, or HHV-6) in the quantifiable range of qPCR assays performed in the Clinical Laboratory Improvement Amendments (CLIA; license 05D1038598) and the College of American Pathologists (CAP; license CAP 2379301)-accredited Stanford Clinical Virology Laboratory and sufficient specimen volume to extract for mNGS and qPCR testing of all 10 viruses. Historical viral load data were not used. For all experiments, total nucleic acids were extracted from 400 μl of plasma using the EZ1 virus minikit version 2.0 on the EZ1 Advanced XL instrument (Qiagen) and eluted in 60 μl of AVE buffer (RNase-free water with 0.04% NaN_3_). The electronic medical record was reviewed for all cases in which Galileo detected a virus that was (i) not concurrently ordered by the clinician at the time of testing and (ii) confirmed by retrospective qPCR testing. Contribution to the patient’s clinical disease was assessed based on the presence of signs and/or symptoms at the time of specimen collection consistent with those of disease caused by the detected virus. If consistent, further evaluation included prior and/or subsequent detection of the virus in question by routine testing during the same clinical episode. In addition, the presence or absence of other laboratory-confirmed diagnoses was reviewed, and if present, it was determined whether the patient responded to directed therapy for that diagnosis.

### Quality control.

The Galileo pipeline includes (i) run-level full-process controls that are taken through the entire workflow from DNA extraction to sequencing and informatics analysis (a negative plasma matrix control and a positive external control containing whole-virus particles of all viruses at a defined level in a negative plasma background), (ii) internal sample normalization controls, and (iii) high- and low-run controls to aid in quantification estimation (whole-virus particles at two defined levels in a negative plasma background).

### Library preparation and sequencing.

Library preparation was performed according to the manufacturer’s protocols (Arc Bio, LLC). In brief, the eluate was concentrated using magnetic beads (Kapa Pure Beads). Enzymatic fragmentation, end repair, and dA-tailing (Arc Bio, LLC) were performed at 37°C for 5 min and then 65°C for 30 min using an Applied Biosystems Veriti thermal cycler (Thermo Fisher Scientific). Subsequent ligation, depletion, and amplification steps also used this instrument. Fragments were ligated using unique dual-index adapters (Arc Bio, LLC) at 20°C for 15 min and purified using magnetic beads (Arc Bio, LLC). Human DNA fragments were depleted using depletion reagents (Arc Bio, LLC) at 45°C for 2 h, followed by 70°C for 15 min. The library was amplified using library amplification primers (Arc Bio, LLC) for 90°C for 30 s, followed by 14 cycles of 98°C for 10 s and 65°C for 75 s, and then 65°C for 5 min. The PCR product was evaluated with a 2% eGel (Thermo Fisher Scientific) for smears ranging from 200 to 900 bp and purified using magnetic beads (Kapa Pure Beads). Libraries were quantified using a Qubit fluorometer (Thermo Fisher) and Bioanalyzer (Agilent) and pooled equally using a tool provided by Arc Bio. The resulting pool was quantified using a qPCR library quantification kit (Roche) on the Applied Biosystems 7900HT real-time PCR system (Thermo Fisher Scientific) prior to sequencing on the NextSeq 500 platform (Illumina).

For the clinical samples tested at the Stanford Clinical Virology Laboratory, an initial calibration run was performed testing the multianalyte mixture of whole-virus particles at viral loads of 0, 100, 1,000, 5,000, 10,000, and 100,000 copies/ml or IU/ml plasma, in triplicate. Positive (10,000 copies/ml multianalyte mix in plasma), negative (plasma), high-run (100,000 copies/ml multianalyte mix in plasma), and low-run (5,000 copies/ml multianalyte mix in plasma) controls provided by Arc Bio, LLC, were processed alongside each run of 10 clinical samples (5 batches of 10 samples plus 4 controls total). Eighteen (calibration) or 14 (clinical samples) libraries were sequenced per high-output NextSeq run.

### Bioinformatics analysis.

System-level NextSeq quality metrics, including error rate, cluster density, and cluster passing filter, were evaluated according to the manufacturer’s recommendations (Illumina). The sample sheet was downloaded from the Galileo Analytics Web application (Arc Bio, LLC), and demultiplexing was then performed using bcl2fastq 2.20 with default parameters and no lane splitting. The resulting FASTQ files were uploaded and analyzed using the Galileo Analytics Web application, which automatically processes uploaded FASTQ files from both samples and controls and produces a quality control (QC) report and a pathogen identification (ID) report for each library.

Galileo uses an alignment module and scores reads based on complexity, uniqueness, and alignment to the targeted DNA viruses. Raw data from the uploaded FASTQ files are transformed into a proprietary signal value, taking into account complexity, unique placement, and alignability of mapped reads. This value normalizes read counts across libraries, normalizes for differing genome lengths, and normalizes for technical bias via the synthetic spiked-in normalization controls. The final result is a reported “signal,” or evidence value, related to genomic depth and likelihood of observing nucleic acid of the viruses in the sample, including nucleic acid belonging to nonconfounding genomic regions. The signal value enables quantitative evaluation of viral load via a standard calibration curve and the ability to compare results across different libraries and different runs.

Run-level quality control criteria were defined using the negative matrix and positive external controls. The negative matrix control was expected to yield no signal for each of the target viruses. The external positive control (10,000 IU or copies per ml) was expected to yield signal values within predefined ranges based on the manufacturer’s internal QC data (Arc Bio, LLC). In addition, library-level quality control metrics were reported in the QC report. All libraries, including the run-level controls, were recommended to be sequenced to a minimum of 30 million total reads and a minimum of 250,000 nonhuman reads, with >80% of bases having a Q score of 30 or greater and >85% of bases having a Q score of 20 or greater, according to the Illumina NextSeq 500 system specifications. GC content was expected to be 35% to 50% due to the majority of the DNA being of human origin. In addition, the synthetic normalization controls were expected to yield signal values in a predefined range based on the manufacturer’s internal QC data (Arc Bio, LLC). For evaluation of the clinical specimens, a minimum of 250,000 nonhuman reads or at least 30 million reads per library were required for subsequent analysis.

FASTQ files from clinical samples in which the Galileo and qPCR results were discrepant were analyzed using an alternative metagenomic NGS analysis pipeline (25).

### Evaluation of analytical performance characteristics.

**(i) Limit of detection/lower limit of quantitation.** NGS libraries were prepared from virus-negative plasma matrix spiked with a multivirus panel at concentrations of 0, 1, 20, 40, 75, 150, 300, 1,200, and 10,000 IU or copies/ml, with 3 or 18 replicates at each concentration. All libraries were processed through the Galileo analytical pipeline for virus identification, and a probit regression model was generated to determine the limit of detection (LoD), or the lowest concentration at which each individual virus was detected in 95% of replicates (signal in 3/3 or 17/18 of replicates at a specific viral load).

The lower limit of quantitation (LLoQ) was calculated to be the recovered viral load, which was (i) greater than or equal to the limit of detection and (ii) reproducible across sequencing runs with a percent coefficient of variation (% CV) less than or equal to 35%.

**(ii) Linearity.** NGS calibration libraries were prepared from virus-negative plasma matrix spiked with a multivirus panel at concentrations of 1, 150, 1,000, 5,000, 10,000, and 100,000 IU or copies/ml, with 3 to 5 replicates at each concentration. All libraries were processed through the Galileo analytical pipeline to generate a virus-specific quantitation signal. Virus-specific linear regression models were generated using the calibration libraries. A coefficient of determination (*R*^2^) was generated from these models to assess the correlation of input viral load with signal. These models then served as the calibration curves to convert signal to international units or copies per milliliter for each virus and therefore provide estimates of the recovered viral load from each run in the probit.

**FIG 2:**
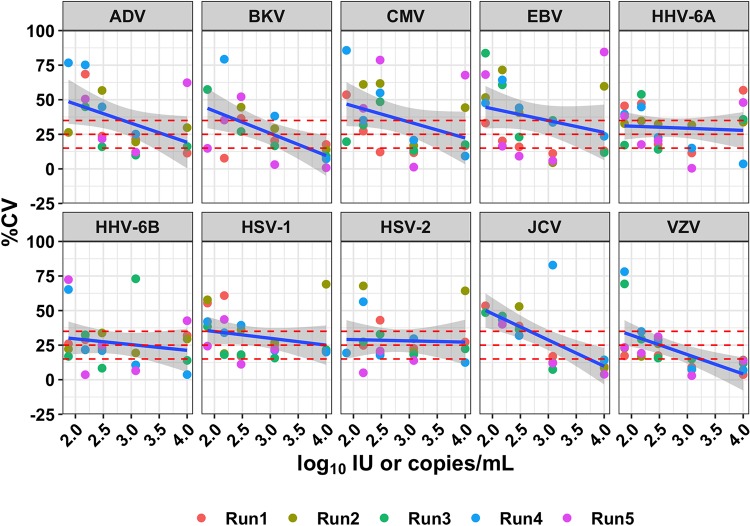
Interrun precision as a function of concentration. Concentrations are shown in log_10_ international units or copies per milliliter; % CV was calculated based on the non-log_10_-transformed values. Points are colored by sequencing run, and the shaded area represents the 95% confidence interval. Dashed horizontal lines indicate commonly used acceptance thresholds for LoD and LLoQ in PCR-based assays (15 and 25% CV, respectively) and the LLoQ for this assay (35% CV).

**(iii) Precision.** Precision was evaluated using three categories of replicate recovered viral load comparisons, interrun, operator interrun, and operator intrarun, and was expressed as the percent coefficient of variation. Interrun precision (Fig. 2; see also Fig. S2 in the supplemental material) was calculated as the ratio of run standard deviation of signal for a virus at a specific load to the mean signal of a virus at the same viral load across all analytical runs. Trend lines and 95% confidence intervals for interrun precision were generated using data across all runs and all viral load points. Operator interrun precision (Fig. S3) was calculated as the ratio of the standard deviation of signal for a virus at a specific load in a run to the mean signal of a virus at the same viral load across all runs for each set of operator-generated sequencing libraries. Trend lines and 95% confidence intervals for operator interrun precision were generated using data across all runs and all viral load points. Operator intrarun precision was calculated as the ratio of the standard deviation of signal for a virus at a specific load to the mean signal of a virus at the same viral load within the run, for each run, across both operators. Trend lines and 95% confidence intervals for operator intrarun precision were generated using data across all runs with operator-matched viral load points.

### qPCR assays.

The RealStar BKV PCR (Altona), artus CMV RCQ MDx (Qiagen), artus EBV PCR (Qiagen), Real Star JCV 1.0 (Altona), and artus HSV-1/2 (Qiagen) qPCR assays were performed according to the manufacturers’ instructions. The ADV and HHV-6 qPCR assays were laboratory-developed tests; additional details regarding these tests are outlined in the Supplemental Methods. The respective LLoQ and 95% LoD for each assay in plasma are as follows: ADV, 120 copies/ml and 97 copies/ml; BKV, 200 IU/ml and 66 IU/ml; CMV, 135 IU/ml and 51 IU/ml; EBV, 100 IU/ml and 70 IU/ml; HHV-6, 1,000 copies/ml and 962 copies/ml; and JCV, 150 copies/ml; 85 copies/ml.

### Statistical analysis of clinical data.

Total, positive, and negative percent agreement and κ coefficients were calculated to assess the qualitative agreement between NGS and qPCR. Confidence intervals for indices of positive and negative agreement were calculated as in Graham and Bull (26). Quantitative agreement between assays was evaluated using Passing-Bablok regression and Bland-Altman plots. Statistical analysis was performed with R version 3.3.3 software (RStudio version 1.1.383).

### Data availability.

Sequencing data that support the findings of this study (with human reads removed) have been deposited in the NCBI SRA and can be accessed with the BioProject identifier PRJNA565681.

## RESULTS

### Analytical evaluation: limit of detection, limit of quantitation, and linearity.

The LoD was determined using probit analysis for each of the 10 DNA viruses across 3 to 18 replicates at 8 concentrations ranging from 0 to 10,000 (in copies per milliliter or international units per milliliter, depending on the virus), at a median sequencing depth of 38.5 million reads. The LoD ranged from 14 to 191 copies/ml (Table 1). Viruses with smaller genomes had slightly higher LoDs than did viruses with larger genomes. The probit curves are shown in Fig. S1.

**TABLE 1:** LoD and LLoQ for the 10 viruses tested

Virus	Genome size (kbp)	Limit of detection (95% recall)	Log_10_ limit of detection (95% recall)	Lower limit of quantitation (35% CV)	Log_10_ lower limit of quantitation (35% CV)
ADV	35.5	79 copies/ml	1.9	583 copies/ml	2.77
BKV	5.1	191 copies/ml	2.29	629 copies/ml	2.80
CMV	23.5	78 IU/ml	1.9	577 IU/ml	2.76
EBV	177.3	24 IU/ml	1.39	661 IU/ml	2.82
HHV-6A	156.9	14 copies/ml	1.15	517 copies/ml	2.71
HHV-6B	161.6	14 copies/ml	1.15	540 copies/ml	2.73
HSV-1	152.2	24 copies/ml	1.39	473 copies/ml	2.68
HSV-2	154.7	24 copies/ml	1.39	595 copies/ml	2.78
JCV	5.1	87 copies/ml	1.94	580 copies/ml	2.76
VZV	124.9	24 copies/ml	1.39	442 copies/ml	2.65

**FIG 3:**
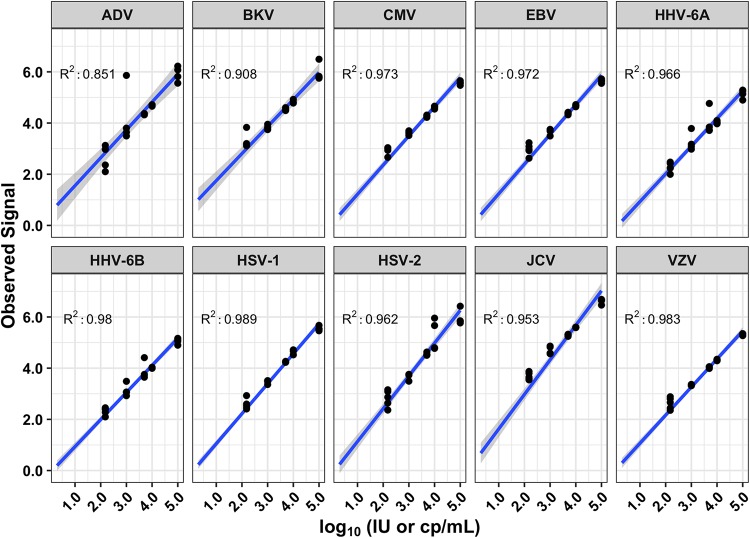
Linearity of the 10 viruses, with associated *R*^2^ values. Concentrations are expressed in log_10_ international units or copies per milliliter, depending on the virus.

The LLoQ, assessed at 35% CV, ranged from 442 copies/ml (VZV) to 661 IU/ml (EBV) (Table 1). Linearity was observed for all viruses in the tested range from the LLoQ to 100,000 IU/ml or copies/ml, which was the highest concentration tested (Fig. 3). *R*^2^ values ranged from 0.85 to 0.98 within the linear range of the assay. The appearance of several outliers, particularly in the ADV plot, likely arises from the stochastic nature of mNGS, which affects how the signal is calculated based on which fragments are recovered, combined with the multistep nature of the protocol.

### Precision.

Precision was evaluated using the libraries prepared for the LoD experiments. A total of 117 libraries were prepared by two different operators and sequenced across multiple sequencing runs (see Materials and Methods). Libraries of the same concentration prepared by the same operator and sequenced on the same sequencing run or on different sequencing runs were used to analyze the intrarun and interrun reproducibility, respectively (Fig. 2 and S2 to S5). Libraries of the same concentration prepared by different operators were used to analyze interoperator reproducibility. Although viral loads are presented as log_10_-transformed concentrations, the % CV was calculated on the non-log_10_-transformed values, as it has been seen to be a closer approximation of the inherent variability of assay signal (27).

### Clinical specimens.

Fifty plasma samples from immunocompromised patients with the presence of at least one DNA virus (ADV, BKV, CMV, EBV, or HHV-6) known from prior clinical testing were tested by mNGS and virus-specific qPCR. Patient characteristics are summarized in Table 2.

**TABLE 2:** Patient characteristics

Characteristic^*a*^	Value
Age (median [range]) (yr)	39.7 (0.5–78.1)
Sex (no. [%])	
Male	26 (52.0)
Female	24 (48.0)
Immunocompromised status (no. [%])	
Transplant	
HCT	24 (48.0)
Kidney	9 (18.0)
Liver	2 (17.0)
Malignancy	
Leukemia	3 (6.0)
Lymphoma	6 (12.0)
HLH	2 (4)
Other^*b*^	4 (8)

a HCT, hematopoietic cell transplant; HLH, hemophagocytic lymphohistiocytosis.

b Ulcerative colitis or chronic fatigue syndrome.

### Clinical evaluation.

**(i) Qualitative.** All samples and controls produced libraries with appropriately sized fragments and were of sufficient concentration to generate library pools for sequencing. All external controls met the manufacturer’s criteria for acceptance. One sample failed sequencing, with only 8,318 total reads and 327 nonhuman aligned reads, and was removed from subsequent analysis. The median sequencing depth was 55,008,780 (range, 18,449,908 to 254,959,658) reads per sample. The median number of nonhuman reads sequenced was 2,394,280 (range, 379,413 to 29,268,082 reads). Note that the sample with only 18,449,908 total reads had 379,413 nonhuman reads, meeting the 250,000-read threshold.

The total percent agreement of mNGS and qPCR was 89.2% (306/343), with a κ statistic of 0.725, demonstrating good agreement between assays. Overall, the positive percent agreement was 84.9% (73/86), and the negative percent agreement was 90.7% (233/257). Among specific viruses, the positive percent agreement ranged from 63.6% (BKV) to 100% (CMV, EBV, ADV, and HSV-1/2), and the negative percent agreement ranged from 80.0% (CMV) to 100% (ADV and BKV) (Table 3). There were 13 samples that were negative by mNGS but positive by PCR; in 100% (13/13) of these samples, the viral load was below the qPCR quantifiable range. Furthermore, 100% (13/13) of these viruses were also not detected by the alternative sequence analysis pipeline.

**TABLE 3:** Qualitative performance of Galileo compared with qPCR^*a*^

Virus(es) tested by Galileo, result	No. with qPCR result:		Positive % agreement (95% CI)	Negative % agreement (95% CI)
	+	−		
All viruses, +	73	24^*b*^	84.9 (77.3–92.5)	90.7 (87.1–94.2)
All viruses, −	13	233		
Adenovirus, +	11	0	100 (100–100)	100 (100–100)
Adenovirus, −	0	38		
BK virus, +	14	0	63.6 (43.5–83.7)	100 (100–100)
BK virus, −	8	27		
Cytomegalovirus, +	14	7	100 (100–100)	80 (66.7–93.3)
Cytomegalovirus, −	0	28		
Epstein-Barr virus, +	14	5	100 (100–100)	85.7 (74.1–97.3)
Epstein-Barr virus, −	0	30		
Human herpesvirus 6, +	12	4	75 (53.8–96.2)	87.9 (76.7–99)
Human herpesvirus 6, −	4	29		
JC virus, +	6	7	85.7 (59.8–100)	83.3 (72.1–94.6)
JC virus, −	1	35		
HSV-1/2, +	2	1	100 (100–100)	97.9 (93.7–100)
HSV-1/2, −	0	46		

a Positive and negative percent agreement were calculated using qPCR as a reference.

b Of the 24 viruses detected solely by mNGS, 7 were confirmed to be positive by an alternative analysis pipeline. These included CMV (*n* = 1), EBV (*n* = 3), HHV-6 (*n* = 2), and HSV (*n* = 1).

mNGS also detected 24 viruses that were not detected by qPCR, including CMV (*n* = 7), EBV (*n* = 5), HHV-6 (*n* = 4), JCV (*n* = 7), and HSV-1/2 (*n* = 1). For CMV, EBV, HHV-6, and HSV-1/2, 88.2% (15/17) were predicted by mNGS to have a low viral load (<2.0 log_10_ copies or IU/ml). mNGS predicted viral loads of >2.0 log_10_ copies or IU/ml in two samples, CMV at 3.58 log_10_ IU/ml and HSV at 2.82 log_10_ copies/ml, that were reproducibly undetectable by qPCR. Of the seven samples in which JCV DNA was detected solely by mNGS, 100% (7/7) were positive for BKV by qPCR. Furthermore, mNGS also called these 7 samples positive for BKV. Overall, only 29.2% (7/24) of these viruses were detected by the alternative sequence analysis pipeline, including CMV (*n* = 1), EBV (*n* = 3), HHV-6 (*n* = 2), and HSV (*n* = 1).

Evaluation of viruses detected by mNGS that were (i) not concurrently ordered by the clinician at the time of testing and (ii) confirmed by retrospective qPCR testing revealed the following 9 additional viruses in 7 patients (4 HCT and 3 oncology): 2 HSV-1/2, 1 HHV-6, 2 BKV, and 4 JCV. Based on a review of the medical records, the detection of these additional viruses by mNGS was, in 8 out of 9 cases, determined to be unlikely to have contributed to the patient’s clinical disease. In one patient, BKV hemorrhagic cystitis had been diagnosed 1 month prior and was known to be resolving at the tested time point, which was confirmed by mNGS. While Galileo may also quantitate VZV, this virus was not detected in the clinical samples tested.

### Clinical evaluation.

**FIG 4:**
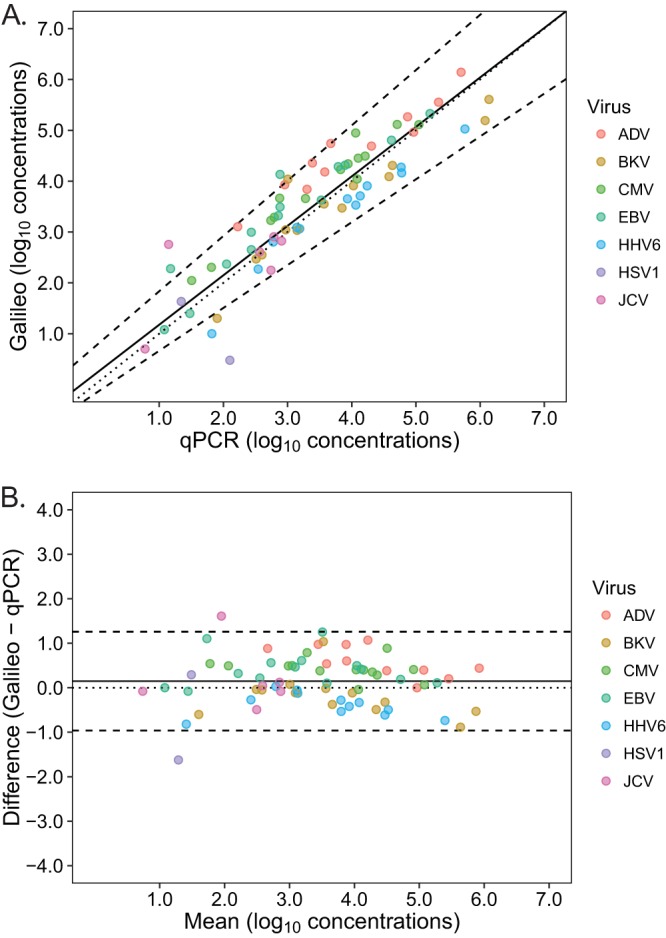
Quantitative agreement of Galileo and qPCR. (A) Passing-Bablok regression resulted in the following regression line of *y* = 0.95*x* + 0.45, with 95% confidence intervals of the slope (0.85 to 1.05) and intercept (0.05 to 0.90). The regression line (solid line), line of identity (dotted line), and 95% confidence intervals (dashed lines) are displayed. (B) Bland-Altman plots demonstrated a mean difference of 0.28 log_10_ concentration (Galileo − PCR), with 95% limits of agreement of −0.62 to 1.18. The 95% limits of agreement (dashed lines) and zero line (dotted line) are also shown.

**(ii) Quantitative.** A calibration run was performed testing the multianalyte mixture of whole-virus particles at viral loads of 0, 100, 1,000, 5,000, 10,000, and 100,000 copies/ml or IU/ml plasma, in triplicate, to produce a standard curve for each virus (Table S1). These curves were then used to calculate viral loads for the clinical samples tested. To investigate the quantitative agreement between Galileo and qPCR, the log_10_ copies/ml or IU/ml of clinical samples that were quantifiable by qPCR were plotted against one another, and a Passing-Bablok regression was performed. This analysis resulted in a regression line of *y* = 0.95*x* + 0.45, with 95% confidence intervals of the slope (0.85 to 1.04) and intercept (0.05 to 0.90), indicating that overall, mNGS displayed no proportional bias or systematic bias compared with qPCR (Fig. 4A). Next, the differences in log_10_ concentrations were plotted against the average values to generate a Bland-Altman plot. The mean difference was +0.28 log_10_ concentration (Galileo − qPCR), with 95% limits of agreement of −0.62 to 1.18 (Fig. 4B). Passing-Bablok regression and Bland-Altman plots for each individual virus are found in Fig. S6.

## DISCUSSION

This study evaluated the analytical and clinical performance characteristics of the Galileo Pathogen Solution mNGS pipeline for quantitation of 10 transplant-associated DNA viruses using reference material and clinical specimens from immunocompromised patients. Overall, mNGS demonstrated qualitative and quantitative performance comparable to those of single-target, standard-of-care qPCR assays. The quantitative accuracy and precision of the Galileo approach are unique in the commercial metagenomics space, and these features, combined with the potential for expansion to additional targets, provide the framework for a comprehensive assay for the diagnosis and monitoring of infectious diseases.

The most important aspect of this study was the validation of the Galileo viral load prediction capability for the quantitation of viral DNA from mNGS sequencing data. To our knowledge, the use of mNGS for determination of viral loads has not been previously demonstrated. Though a recent study reported a correlation between an mNGS-based readout and qPCR for a small number of samples, viral load was not calculated from the sequencing data (28). mNGS-based quantitation is challenging from many perspectives; for example, variation in human background nucleic acid and technical biases can affect the viral sequencing depth, which affects obtained target reads. The Galileo viral load prediction capability addresses these challenges by taking into account the complexity, unique placement, and alignability of mapped reads, and the generated signal value normalizes read count across libraries, differing genome lengths, and technical bias via synthetic spiked in normalization controls. As such, the analytical evaluation of Galileo demonstrated LoDs, precision analyses, and linear ranges consistent with qPCR.

Critically, the clinical study, performed independently of the manufacturer at an academic medical center, confirmed the similar performance of mNGS compared to qPCR. Quantitatively, Passing-Bablok regression showed no overall systematic or proportional bias, and Bland-Altman analysis revealed a slight positive mean difference (+0.28 log_10_ concentration), even when including samples below the LLoQ of mNGS. This result suggests that the approach used for calculating the LLoQs for the mNGS assay, using a coefficient of variation cutoff of 35%, which is intermediate between typical qPCR cutoffs (29) and previous mNGS approaches (28), may be overly conservative, despite the variability observed in the signal value in the precision experiments. Nevertheless, most qualitative discrepancies occurred in specimens in which the viral load was below the LLoQ of either assay. These low-level signals may simply represent assay noise; however, they may also indicate early viral replication or latent/persistent viral genomes, both of which are of uncertain clinical significance. Notably, if only results in the quantifiable range of both mNGS and qPCR were considered, the total percent agreement was 99.3% (294/296), the positive percent agreement was 100% (61/61), and the negative percent agreement was 99.1% (233/235). In addition, there were specificity concerns in the original bioinformatics analysis. Of the viruses detected by Galileo that were not detected by qPCR, only 29.2% (7/24) were detected by an alternative sequence analysis pipeline (25). The viruses detected only by Galileo included CMV (*n* = 6), EBV (*n* = 2), HHV-6 (*n* = 2), and JCV (*n* = 7). Of these viruses, JCV was of particular concern, as all of these specimens were BKV positive by both qPCR and Galileo (*n* = 7). However, when the Galileo Analytics pipeline was updated for analysis of only nonconfounding genomic regions, the JCV false positives were resolved. Further updates to the Galileo Analytics pipeline are required to address the false positives observed for other viruses.

Though Galileo provides qPCR-comparable detection and quantitation of transplant-associated DNA viruses through the incorporation of a proprietary viral load prediction capability, at present, this method is not yet expected to supplant qPCR for routine virus monitoring of immunocompromised patients. In particular, qPCR remains less costly and less laborious, and it provides a more rapid turnaround time than this quantitative mNGS approach. For example, the Galileo workflow takes approximately 48 h to complete, of which ∼20 h is sequencing, while qPCR requires ∼4 to 6 h, including extraction, reaction setup, PCR, and analysis. mNGS typically also requires technical and computational expertise to adopt, and many clinical microbiology and virology laboratories do not have personnel with the necessary skill sets. Furthermore, process controls, validation strategies, and QC criteria for both the wet and computational components must be defined (30). This effort becomes even more complicated for mNGS tests that aim to detect a large number of organisms, including common laboratory contaminants (28, 31). Galileo overcomes several of these limitations by providing the process and standard controls required to perform the assay, reagents, software, and quantitative reporting of a targeted set of organisms. In the short term, the myriad challenges of mNGS assays remain a barrier to routine use in diagnostic infectious disease laboratories; however, widespread implementation of Galileo and other mNGS approaches for clinical use will be made possible by the ongoing development of solutions to automate and simplify library preparation, as well as innovations in methods to reduce sequencing depth without sacrificing sensitivity.

A significant advantage that Galileo has over single-target qPCR assays is the ability to detect and accurately quantitate coinfecting viruses in a single test. In contrast to the Galileo data presented here, previous work in this area using PCR coupled with real-time capillary electrophoresis (22) and multiplex targeted sequencing (23) demonstrated reduced clinical sensitivity compared to qPCR. In addition, these assays were not evaluated for their quantitative performance characteristics. Nevertheless, the presence of virus coinfections in transplant recipients is well described; for example, a study of 156 HCT recipients found that one-third had two or more viruses detected in plasma by day 180 posttransplant (32). Importantly, virus coinfections in transplant recipients may lead to increased complications (33). mNGS detected 9 additional coinfecting viruses (2 HSV-1/2, 1 HHV-6, 2 BKV, and 4 JCV) in 7 patients (4 HCT and 3 oncology) where targeted testing was not ordered at the time of initial monitoring. Though a chart review revealed no evidence that the coinfecting viruses contributed to the clinical outcome in these particular cases, future prospective, randomized controlled trials of mNGS compared to standard infectious diseases testing may be instrumental in demonstrating the unique clinical utility of quantitative mNGS approaches (34).

In addition to its retrospective nature and the selection of archived clinical specimens for the purposes of method comparison rather than analysis of clinical outcomes, other limitations of this study included a small sample size that precluded virus-level quantitative analysis of clinical specimens and the absence of specimens positive for other viruses quantitated by Galileo (VZV). Furthermore, it is important to note that this study described the performance characteristics of a precommercial, research use only (RUO) version of Galileo. As various improvements are made, such as those described for the Galileo Analytics pipeline, evaluation of future versions would also be warranted.

In conclusion, Galileo is a complete mNGS sequencing reagent and bioinformatics pipeline with a unique viral load prediction capability that demonstrates performance comparable to that of singleplex qPCR but with the key advantage of allowing for the simultaneous detection and quantitative analysis of 10 transplant-related DNA viruses (ADV, BKV, CMV, EBV, HHV-6A, HHV-6B, HSV-1, HSV-2, JCV, and VZV). In its current form, Galileo may enable critical outcome studies of virus coinfections in immunocompromised patients.

## Supplementary Material

Supplemental file 1
