# Supplementary material for: Metagenomic Next-Generation Sequencing for Identification and Quantitation of Transplant-Related DNA Viruses
Source: J Clin Microbiol. 2019 Nov 22;57(12):e01113-19. doi: 10.1128/JCM.01113-19 (PMC6879295; doi:10.1128/JCM.01113-19)
Supplement: Supplemental file 1 [file JCM.01113-19-s0001.pdf]

Supplemental Table

Table S1. Clinical Calibration Run

Virus	Slope	Y-intercept	r^2
ADV	1.13 ± 0.04	-8.09 ± 0.15	0.99
BKV	1.17 ± 0.08	-7.89 ± 0.34	0.95
CMV	1.11 ± 0.08	-7.60 ± 0.30	0.94
EBV	1.07 ± 0.10	-7.94 ± 0.37	0.91
HHV-6A	1.01 ± 0.02	-7.91 ± 0.09	0.99
HHV-6B	1.08 ± 0.03	-8.16 ± 0.11	0.99
HSV-1	1.10 ± 0.08	-8.13 ± 0.29	0.94
HSV-2	1.16 ± 0.10	-8.25 ± 0.36	0.92
JCV	0.95 ± 0.04	-6.13 ± 0.15	0.98
VZV	0.97 ± 0.06	-8.21 ± 0.23	0.96

Best-fit parameters of linear regression between log_{10} viral load and log_{10} signal using spiked samples at known concentrations tested by the Galileo pipeline.

Supplemental Figures

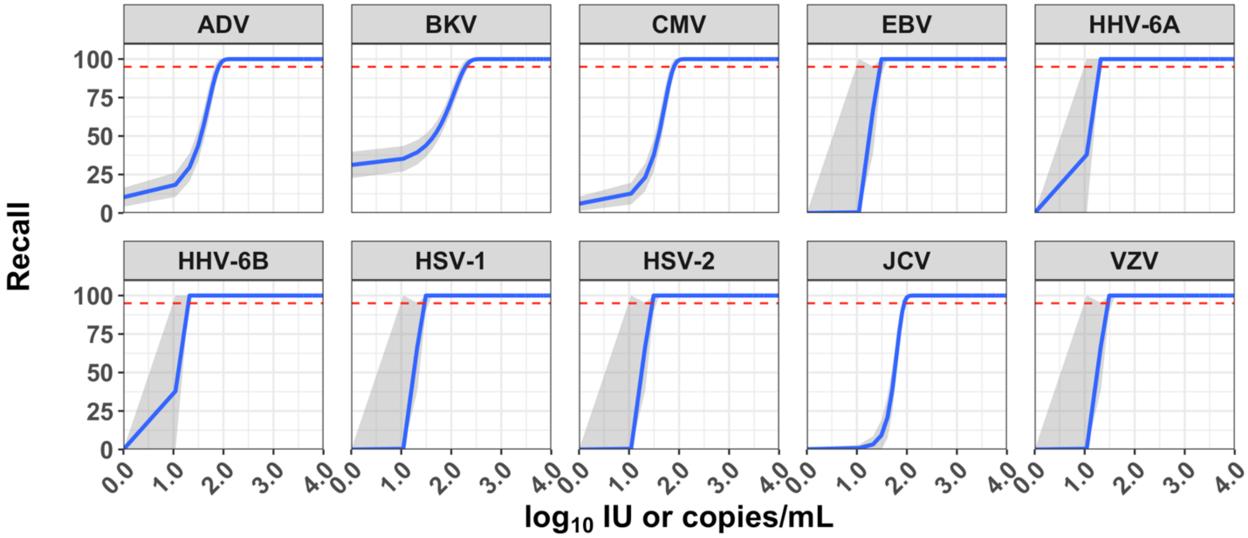

Figure S1: Probit curves for the 10 viruses tested in the LoD experiment. Shaded areas represent the 95% confidence intervals. Dashed horizontal line represents 95% recall.

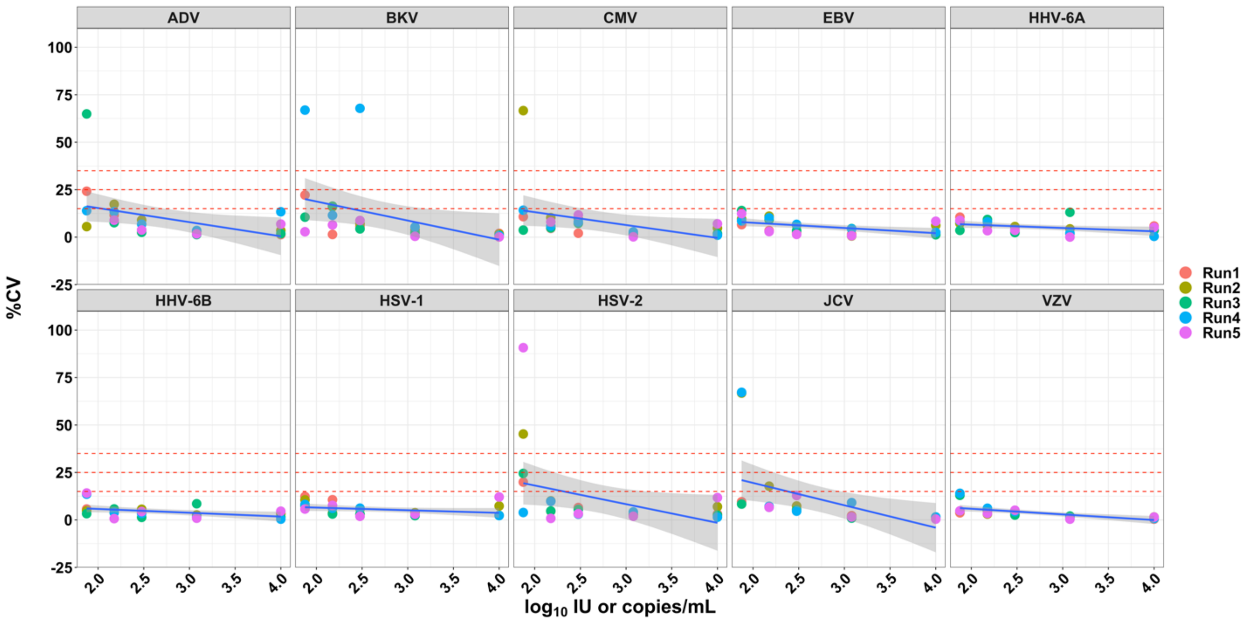

Figure S2: Inter-run $\log_{10}$ precision. Concentrations are shown in $\log_{10}$ IU or copies/mL, and the % CV was calculated based on $\log_{10}$-transformed values. Points are colored by the sequencing run, and the shaded areas represent the 95% confidence intervals. Dashed horizontal lines indicate commonly used acceptance thresholds for LoD and LLoQ in PCR-based assays (15 and 25% CV, respectively) and the LLoQ for this assay (35% CV).

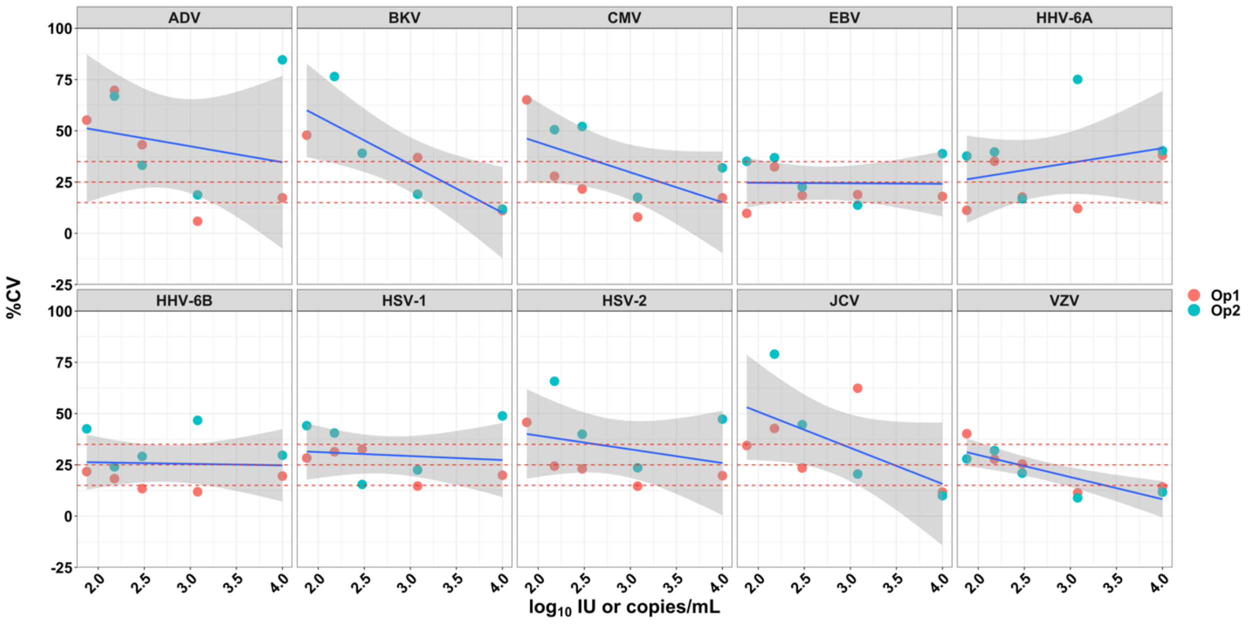

Figure S3: Operator inter-run precision. Concentrations are shown in log_{10} IU or copies/mL, and the % CV was calculated based on log_{10}-transformed values. Points are colored by operator-generated libraries (Op1 is operator 1 and Op2 is operator 2), and the shaded areas represent the 95% confidence intervals. Dashed horizontal lines indicate commonly used acceptance thresholds for LoD and LLoQ in PCR-based assays (15 and 25% CV, respectively) and the LLoQ for this assay (35% CV).

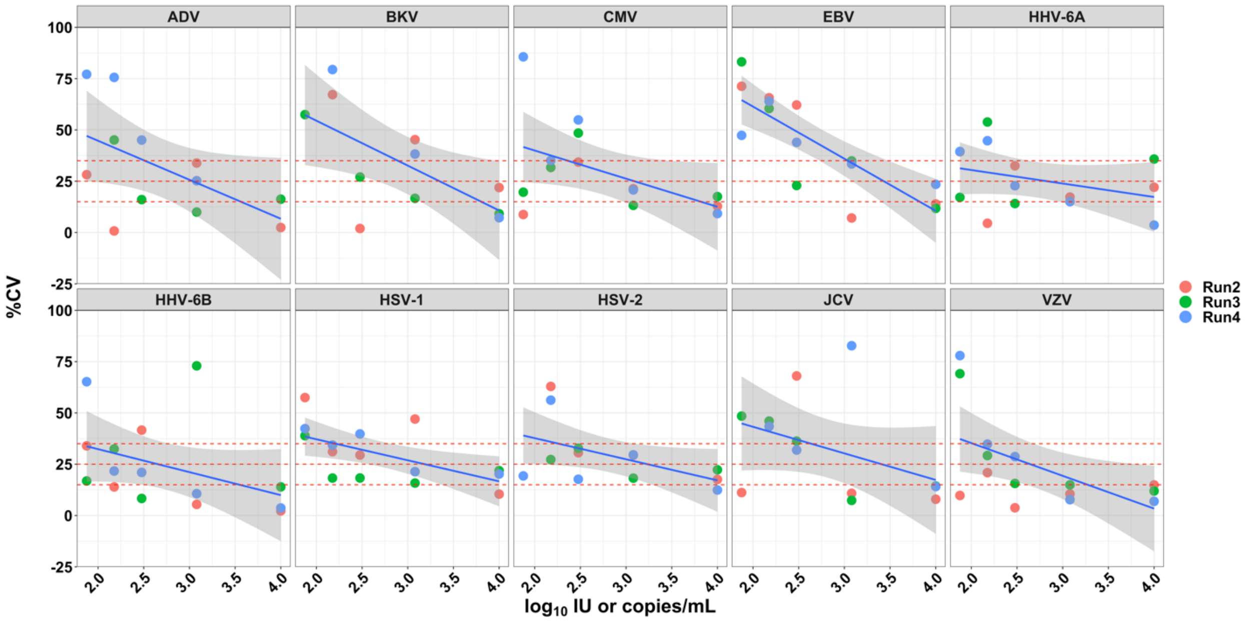

Figure S4: Operator-matched intra-run precision. Concentrations are shown in $\log_{10}$ IU or copies/mL, and the % CV was calculated based on non-$\log_{10}$-transformed values. Points are colored by the sequencing run, and the shaded areas represent the 95% confidence intervals. Dashed horizontal lines indicate commonly used acceptance thresholds for LoD and LLoQ in PCR-based assays (15 and 25% CV, respectively) and the LLoQ for this assay (35% CV).

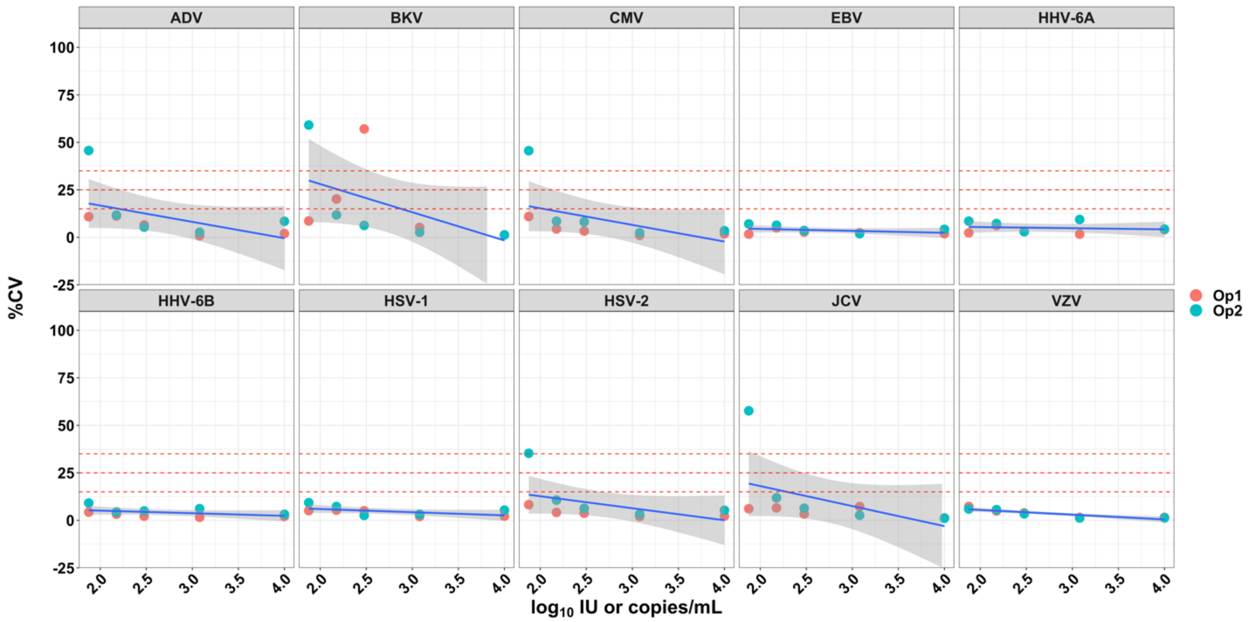

Figure S5: Operator inter-run $\log_{10}$ precision. Concentrations are shown in $\log_{10}$ IU or copies/mL, and the % CV was calculated based on $\log_{10}$-transformed values. Points are colored by the operator generating the libraries, and the shaded areas represent 95% confidence intervals. Dashed horizontal lines indicate commonly used acceptance thresholds for LoD and LLoQ in PCR-based assays (15 and 25% CV, respectively) and the LLoQ for this assay (35% CV).

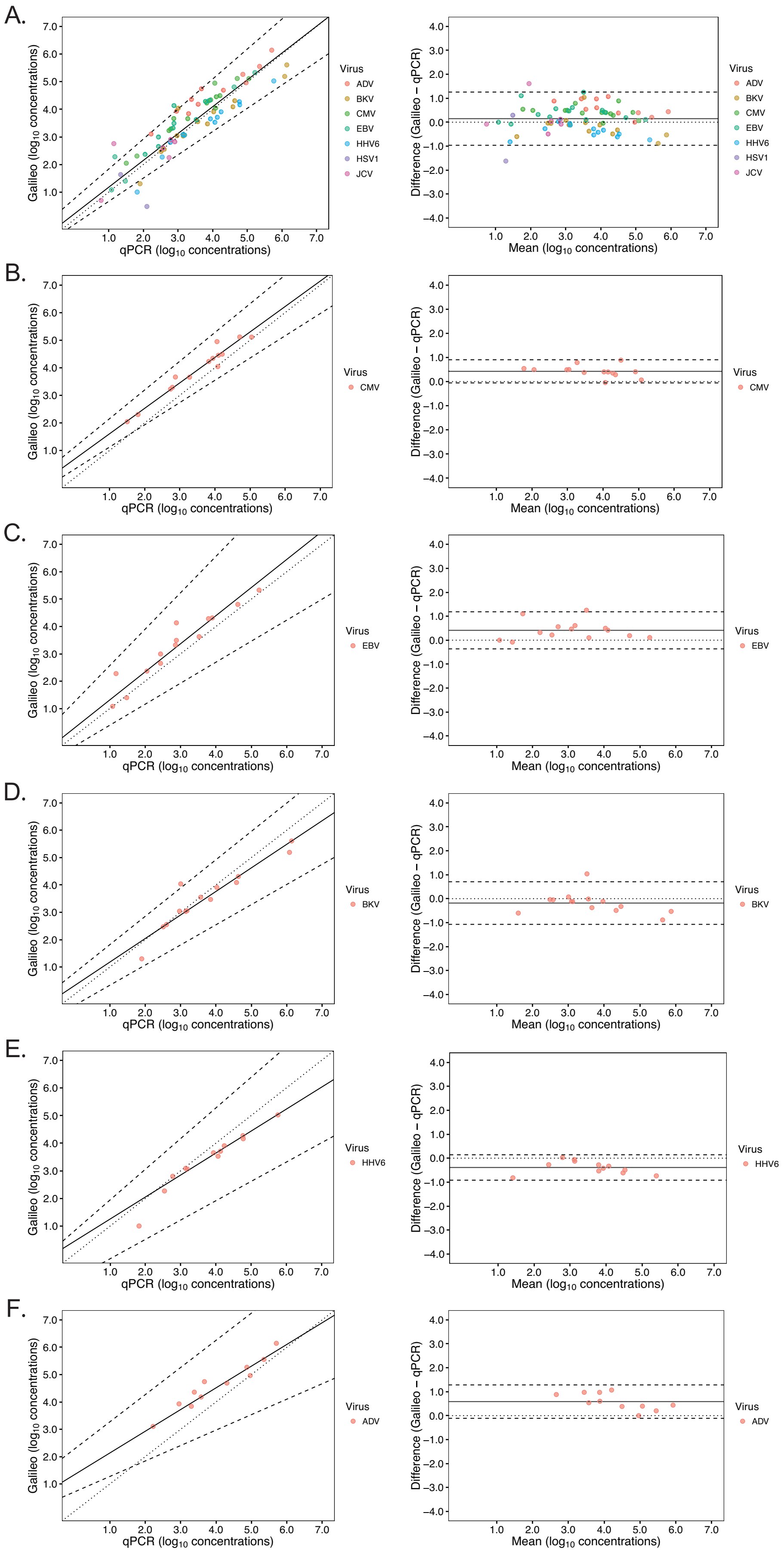

A.
Passing-Bablok
Intercept 0.20, 95%CI (-0.17 to 0.75)
Slope 0.97, 95%CI (0.84 to 1.09)

Bland-Altman
Bias 0.1, 95%CI (-0.96 to 1.26)

B.
Passing-Bablok
Intercept 0.68, 95%CI (0.32 to 1.11)
Slope 0.92, 95%CI (0.81 to 1.04)

Bland-Altman
Bias 0.42, 95%CI (-0.05 to 0.90)

C.
Passing-Bablok
Intercept 0.29, 95%CI (-0.38 to 1.24)
Slope 1.03, 95%CI (0.77 to 1.33)

Bland-Altman
Bias 0.41, 95%CI (-0.36 to 1.19)

D.
Passing-Bablok
Intercept 0.32, 95%CI (-0.40 to 0.78)
Slope 0.86, 95%CI (0.74 to 1.03)

Bland-Altman
Bias -0.18, 95%CI (-1.07 to 0.71)

E.
Passing-Bablok
Intercept 0.46, 95%CI (-0.89 to 0.84)
Slope 0.80, 95%CI (0.70 to 1.11)

Bland-Altman
Bias -0.39, 95%CI (-0.92 to 0.14)

F.
Passing-Bablok
Intercept 1.34, 95%CI (0.71 to 2.28)
Slope 0.79, 95%CI (0.56 to 0.99)

Bland-Altman
Bias 0.59, 95%CI (-0.11 to 1.28)

Figure S6: Quantitative agreement of Galileo and qPCR for individual viruses. Passing-Bablok regression and Bland-Altman plots are shown for all viruses (A), CMV (B), EBV (C), BKV (D), HHV-6 (E), and Adenovirus (F). Each analysis includes the slopes, intercepts, bias, and associated 95% confidence intervals (CI).

Supplemental Methods

ADV quantitative assay

ADV DNA was quantitated using a laboratory-developed real-time PCR assay modified from Huang et al. that amplifies all ADV species (1). The ADV assay was performed using the QuantiFast Pathogen +IC kit on the Rotor-Gene Q instrument (Qiagen). Each reaction was performed using 10 µL of eluate, with a final reaction volume of 25 µL. Primer and probes (both from Biosearch Technologies, Petaluma, CA) targeting the hexon and penton genes were as described previously (1), but with BHQ-1 used as quencher rather than TAMRA. ADV A and ADV F primers were added at final concentrations of 400 nM, whereas primers ADV B, ADV C, and ADV E were added at final concentrations of 280 nM. Final concentrations of probes were 100 nM for ADV A and ADV F probes, 64 nM for ADV B and ADV C probes, and 72 nM for ADV E probes. Cycling conditions were as follows: hold at 95°C for 5 minutes, followed by 45 cycles of 95°C for 15 seconds and 60°C for 30 seconds. Detection was performed in the green (ADV) and yellow (IC) channels; the threshold was set at .04 for the green channel and .07 for the yellow channel. The assay was calibrated using a plasmid containing the hexon region of the ADV 11 (ADV B species) genome.

HHV-6 quantitative assay

HHV-6 DNA was quantitated using a laboratory-developed real-time PCR assay targeting the HHV-6 *U66* gene (QuantiFast Pathogen +IC kit on a Rotor-Gene Q instrument; Qiagen, Germantown, MD). Each reaction was performed using 10 µL of eluate in a final reaction volume of 25 µL. Primers (HHV6Q_FWD: GAACACGTGGGTCAGATAGTTGAT; HHV6Q_REV: CATCGCCGTCACCAAACTT; Eurofins MWG Operon, Louisville, KY) and hydrolysis probes

(HHV6Q probe: FAM-CACGATTGGCTAAAGC-MGB-NFQ; ThermoFisher Scientific, Waltham, MA) were added at final concentrations of 400 nM and 200 nM, respectively. The QuantiFast Pathogen +IC master mix also contains a primer/probe set targeting the IC DNA. Cycling conditions were as follows: hold at 95°C for 5 minutes, followed by 45 cycles of 95°C for 15 seconds and 60°C for 30 seconds. Detection was performed in the green (HHV-6) and yellow (IC) channels; the threshold was set at 0.1 for both channels. The assay was calibrated using a plasmid containing the assay target region of the HHV-6B genome.

Supplemental References

1. Huang ML, Nguy L, Ferrenberg J, Boeckh M, Cent A, Corey L. 2008. Development of multiplexed real-time quantitative polymerase chain reaction assay for detecting human adenoviruses. Diagn Microbiol Infect Dis 62:263-71.
